# Montreal Veterinary Medical Association

**Published:** 1890-05

**Authors:** 


*Montreal Veterinary Medical Association.*—The closing meeting was held on Friday evening, March 28th, in the lecture room of the college. Diplomas were conferred on Messrs. McGlue, Scanlan, Scott, Hayman, Darling, Willyoung, Walsh, Crossman. The prizes were awarded as follows: 1st, M. H. Scanlan; 2d, J. McGlue; 3d, L. E. Willyoung.

The *Society for the Study of Comparative Psychology* held its closing meeting on the same evening, the president, Dr. Mills, in the chair. This brought to a close a series of interesting and instructive meetings, and were well calculated to insure better treatment to the dumb creation. Diplomas were also awarded to the same gentlemen. Prizes for best papers were awarded to: 1st, R. N. Walsh; 2d, L. E. Willyoung.

